# Clade I–Associated Mpox Cases Associated with Sexual Contact, the Democratic Republic of the Congo

**DOI:** 10.3201/eid3001.231164

**Published:** 2024-01

**Authors:** Emile M. Kibungu, Emmanuel H. Vakaniaki, Eddy Kinganda-Lusamaki, Thierry Kalonji-Mukendi, Elisabeth Pukuta, Nicole A. Hoff, Isaac I. Bogoch, Muge Cevik, Gregg S. Gonsalves, Lisa E. Hensley, Nicola Low, Souradet Y. Shaw, Erin Schillberg, Mikayla Hunter, Lygie Lunyanga, Sylvie Linsuke, Joule Madinga, Martine Peeters, Jean-Claude Makangara Cigolo, Steve Ahuka-Mundeke, Jean-Jacques Muyembe, Anne W. Rimoin, Jason Kindrachuk, Placide Mbala-Kingebeni, Robert S. Lushima, International Mpox Research Consortium

**Affiliations:** Ministry of Public Health, Kinshasa, Democratic Republic of the Congo (E.M. Kibungu, T. Kalonji-Mukendi, R.S. Lushima);; Institut National de Recherche Biomédicale, Kinshasa (E.H. Vakaniaki, E. Kinganda-Lusamaki, E. Pukuta, L. Lunyanga, S. Linsuke, J. Madinga, J.-C. Makangara Cigolo, S. Ahuka-Mundeke, J.-J. Muyembe, P. Mbala-Kingebeni);; Cliniques Universitaires de Kinshasa, Université de Kinshasa, Kinshasa (E. Kinganda-Lusamaki, J.-C. Makangara Cigolo, S. Ahuka-Mundeke, J.-J. Muyembe, P. Mbala-Kingebeni);; TransVIHMI (Recherches Translationnelles sur le VIH et les Maladies Infectieuses endémiques et émergentes);; University of Montpellier, French National Research Institute for Sustainable Development, INSERM, Montpellier, France (E. Kinganda-Lusamaki, M. Peeters);; University of California, Los Angeles, California, USA (N.A. Hoff, A.W. Rimoin);; Toronto General Hospital, University Health Network, Toronto, Ontario, Canada (I.I. Bogoch);; University of St. Andrews, St. Andrews, Scotland, UK (M. Cevik);; Yale School of Public Health, New Haven, Connecticut, USA (G.S. Gonsalves);; USDA Agricultural Research Service, Manhattan, Kansas, USA (L.E. Hensley);; University of Bern, Bern, Switzerland (N. Low);; University of Manitoba, Winnipeg, Manitoba, Canada (S.Y. Shaw, E. Schillberg, M. Hunter, J. Kindrachuk)

**Keywords:** mpox, monkeypox virus, MPXV, viruses, sexually transmitted infections, the Democratic Republic of the Congo

## Abstract

We report a cluster of clade I monkeypox virus infections linked to sexual contact in the Democratic Republic of the Congo. Case investigations resulted in 5 reverse transcription PCR–confirmed infections; genome sequencing suggest they belonged to the same transmission chain. This finding demonstrates that mpox transmission through sexual contact extends beyond clade IIb.

Human mpox, caused by monkeypox virus (MPXV), is an emerging zoonotic viral disease first identified in the Democratic Republic of the Congo (DRC) (*1*). MPXV is endemic in multiple regions of Central and West Africa (*2*,*3*). The virus is subclassified into 2 clades: clade I, formerly Congo Basin (Central Africa) clade, and clade II, formerly West African clade. Clade II is further subdivided into 2 subclades, IIa and IIb; subclade IIb was responsible for the 2022 global epidemic (*4*; https://www.who.int/news/item/12-08-2022-monkeypox--experts-give-virus-variants-new-names). Clade I infections are associated with greater disease severity and more pronounced rash and had demonstrated increased human-to-human transmission compared with clade II before the global emergence of clade IIb (*5*). Those difference are likely influenced by factors such as clade-specific genomic differences in host response modifier proteins, exposure type and dose, and vaccination status (https://www.who.int/news/item/12-08-2022-monkeypox--experts-give-virus-variants-new-names). Travel-related and animal importation–related cases have been reported in nonendemic regions (*6*). In 2022, rapid spread of MPXV to new geographic regions resulted in >86,000 confirmed infections in nonendemic regions and declaration of a public health emergency of international concern by the World Health Organization (*7*).

During the 2022 epidemic, >90% of infections were linked to secondary transmission, mainly through sexual contact among men who have sex with men (MSM) (*8*–*11*). The disease appeared to affect younger populations in endemic regions because of increased contact with zoonotic sources; the average age at infection was <25 years, and the case-fatality rate was higher among children. In nonendemic regions, however, the average age at infection was >30 years, and infection occurred predominantly in men (>95%) who self-identify as MSM (>80%) (*10*,*11*). Clinical characteristics during the 2022 epidemic included fever, physical asthenia or lethargy, and lymphadenopathy with high concentrations of papules, pustules, and vesicles on the skin of the genital and perianal organs. Rectal pain, bleeding, and purulent bloody stools were also common (*12*,*13*). A recent case series assessing data from clade IIb infections in persons living with HIV demonstrated that increased disease severity and fatal disease were also linked to CD4 counts (*11*).

Although clade I–related infections can occur through close contacts, including through fomites, transmission through sexual contact has not been reported previously (*14*,*15*). However, given the high sequence homology across MPXV clades and the increased disease severity associated with clade I, clarifying whether sexual contact–related infections occur across MPXV clades is critical. We report a confirmed cluster of clade I–associated mpox associated with sexual contact.

## The Study

An alert was issued by the Kwango Provincial Health Division, DRC, after reports from civil society organizations in March 2023 regarding a resident with skin rashes and pruritus. Teams from the National Programme for the Control of Monkeypox and Viral Hemorrhagic Fevers and the Institut National de Recherche Biomédicale, accompanied by members of the senior team from the Kwango Provincial Health Division, the senior team from the Kenge Health Zone, the provincial team from the National Programme for the Control of HIV, and the provincial civil society representative, conducted an investigation. During the investigation, the national and provincial health teams instituted training for local healthcare clinics to raise awareness of the clinical signs and mode of transmission for mpox and HIV, including sexual contact in MSM.

**Figure 1:**
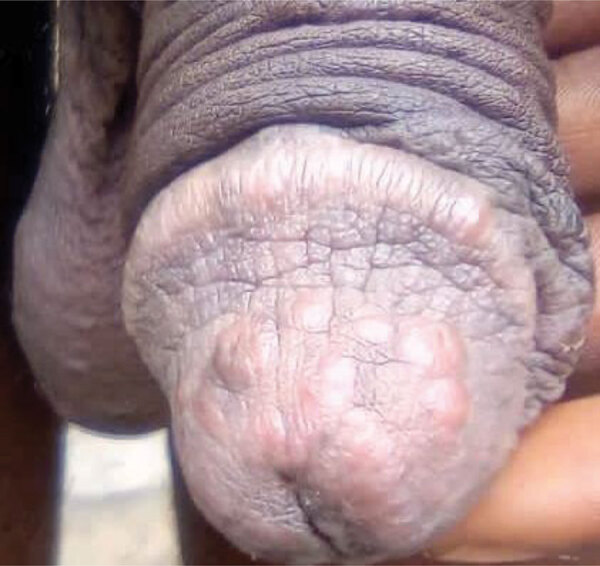
Penile lesions and papules associated with clade I monkeypox virus infection in a man in his late 20s who reported having 2 sexual encounters with a man in Europe 1 week before arriving in Democratic Republic of the Congo.

A man from DRC in his late 20s (case-patient 1) reported having 2 sexual encounters with a man (suspected primary case-patient) in Europe 1 week before returning to DRC. The suspected primary case-patient frequently visited DRC. Case-patient 1 reported that the primary case-patient had general clinical symptoms including genital pruritus, joint pain, and physical asthenia. Nine days after contact, case-patient 1 began experiencing pruritus, vesicular skin eruptions, and genital and perianal ulcerations. He contacted a local civil organization, which initiated a preliminary investigation and alerted the National Programme for the Control of Monkeypox and Viral Hemorrhagic Fevers. He had penile lesions and penile papules (Figure 1); localized redness on the lips and in the oral buccal mucosa were also noted, and he had 1 lesion on the middle finger of the right hand. Blood samples, oropharyngeal swab samples, swab samples from rectal and genital lesions, and swab samples from vesicles on the skin of the penis and pubis were taken.

**Figure 2:**
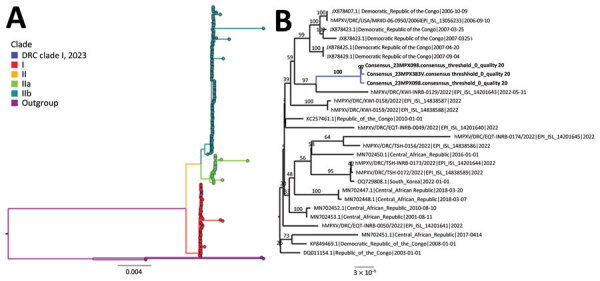
Phylogenetic analysis of MPXV sequences from a cluster of mpox cases described in Kwango Province, Democratic Republic of the Congo. A) MPXV global phylogeny showing that the Kwango Province outbreak cluster belongs to clade I MPXV. B) Phylogenetic analysis of MPXV genome sequences from the reported cases and clade I MPXV sequences from Central Africa. Posterior support values are shown at branch points. DNA was extracted at Institut National de Recherche Biomédicale using a QIAGEN DNA Mini Kit (https://www.qiagen.com) from blood samples and subsequently screened for MPXV with an orthopoxvirus-specific real-time PCR assay. Whole-genome sequencing was attempted on samples from the index case by next-generation sequencing. The library preparation was performed using Illumina DNA Prep with Enrichment (https://www.illumina.com), and the libraries were enriched for MPXV using biotinylated custom probes synthesized by Twist Biosciences (https://www.twistbioscience.com). Note that 23MPX098 (or 23MPX0245V) and 23MPX099 (or 23MPX0245C) are vesicle and crust samples from case-patient 1. Scale bar indicates number of substitutions per site. DRC, Democratic Republic of the Congo; hMPXV, human MPXV; MPXV, monkeypox virus.

Case-patient 1 reported having sexual contact with 9 partners (6 men and 3 women) after arriving in DRC. A prescreening of those contacts identified suspected mpox in 3 sexual contacts (2 men and 1 woman, 30–35 years of age) who had fever and lesions in the genital and perianal region. Blood samples and oropharyngeal swab samples were taken from all 9 partners; additional rectal or vaginal swab samples were taken from the men and woman suspected to be secondary case-patients. Investigation of all sexual contacts yielded an additional 36 sexual contacts. Samples from case-patient 1 and 3 suspected secondary case-patients were confirmed to be MPXV-positive by PCR using samples from vesicles, crusts, or blood. One of the 36 sexual contacts was also PCR-positive for MPXV. We performed viral genome sequencing on PCR-positive samples; phylogenetic analysis showed tight clustering among 3 positive samples, suggesting they belong to the same chain of transmission. The closest related sequence beyond this cluster was a 2022 clade I MPXV sequence from DRC (Figure 2).

We treated the 5 PCR-positive case-patients with supportive care and pain control on an outpatient basis. We performed a follow-up investigation to further investigate transmission chains and identify additional contacts; follow-up was conducted for each contact through individual case report forms. We monitored a total of 120 contacts over 21 days. The contacts were categorized into 3 groups: sexual contacts (n = 5), family members (n = 45), and persons associated with close nonsexual contact (n = 70). None of the persons monitored during the 21 days experienced clinical signs.

## Conclusions

We describe a cluster of clade I MPXV–associated infections in DRC related to sexual contact, which has previously only been described for clade II MPXV. Of note, MPXV transmission through sexual contact is not exclusive to clade IIb and can occur during heterosexual and same-sex contact. This study demonstrates that MPXV infections can occur through additional exposure routes in MPXV-endemic regions and that the current understanding of mpox burden in clade I–endemic regions is based on classical transmission exclusively; recognizing those factors is critical. Our findings highlight additional considerations for MPXV circulation and transmission containment in endemic areas. Thus, increased MPXV surveillance, diagnostic testing access, and equitable access to both vaccines and therapeutics for persons at increased risk for infection are needed for ongoing mitigation strategies. Given the increased disease severity associated with clade I MPXV, the potential implications of sexual transmission on broadening geographic distribution for MPXV across clades I and II must be considered. In addition, long-term immunity to mpox inferred by vaccination is unknown, including the role of mucosal immunity against clade I MPXV infections. This report highlights multiple critical global health considerations that must be addressed. Ongoing support for community engagement and educational efforts focusing on mpox recognition and reporting, including within sexual networks and for specific groups who might suffer from lack of care or experience stigma when seeking care.

Our findings highlight historically unrecognized MPXV transmission through sexual contact and indicate the need for increased routine screening in sexual health clinics in mpox-endemic and nonendemic regions. Population movement and previously unreported routes of transmission could exacerbate global distribution of MPXV, which could be compounded by the lack of routine diagnostic testing or inadequate access to rapid point-of-care testing. In view of this investigation, epidemiologic and genomic surveillance for MPXV, in both endemic and nonendemic regions, should be improved and strengthened.
