# Correction: Vol. 30, No. 1

**DOI:** 10.3201/eid3010.C23010

**Published:** 2024-10

**Keywords:** errata, erratum, correction

Figures were mislabeled and the author list was incorrect in Clade I–Associated Mpox Cases Associated with Sexual Contact, the Democratic Republic of the Congo (E.M. Kibunguet al.). The article has been corrected online (https://wwwnc.cdc.gov/eid/article/30/1/23-1164_article).

